# Untrustworthy Druggists

**Published:** 1887-03

**Authors:** William B. Hazard

**Affiliations:** Prof. Principles and Practice of Medicine, Col. Phys, and Surgs., St. Louis


### UNTRUSTWORTHY DRUGGISTS.

It is a well-known fact that there are druggists in every large city who are not to be trusted with the filling of a prescription that calls for any expensive drug. They come and go, so that at last physicians are compelled to designate certain of the drug fraternity as trustworthy, and insist upon their patients going to these alone for their medical supplies. If they fail to do this, their work is thrown away and their reputations go with the failure of their remedies in critical cases.

A few cases from actual observation and experience will illustrate this better than a volume of argument.

1. Thirty grains of quinine, in three doses, to be taken at hourly intervals, were prescribed for a young man suffering from ordinary intermittent fever. The doses were taken as directed, but no signs of cinchonism were induced, and the disease progressed without change. The same doses, in "Warner's sugar-coated pills," were ordered with the effect of inducing well-marked cinchronism with cure of the disease.

2. In a case of profuse menorrhagia, one ounce of fluid extract of ergot was ordered, with directions to take a fluid drachm every hour until the hemorrhage ceased. The entire amount was taken without result. An ounce of "Squibb's fluid extract of ergot" was ordered—same directions—and the flooding ceased after the second dose.

3. Four ounces of a mixture of bromide of potassium and chloral, each an ounce, with tincture of hyoscyamus and fluid extract of cannabis indica, in appropriate doses, were ordered, with directions to take one teaspoonful every hour until sleep should be induced. An ugly, muddy mixture was received, which produced nausea and headache, but no sleep.

A similar prescription, instead of the above extemporaneous officinal combination, was ordered, only "Battle's BROMIDIA" was designated, which induced refreshing sleep after a few doses of from twenty to thirty drops had been taken. [*Extract from an article in the Dec. Med. Brief, by* William B. Hazard, *Prof. Principles and Practice of Medicine, Col. Phys. and Surgs., St. Louis.*

